# Shared diagnostic genes and potential mechanism between PCOS and recurrent implantation failure revealed by integrated transcriptomic analysis and machine learning

**DOI:** 10.3389/fimmu.2023.1175384

**Published:** 2023-05-16

**Authors:** Wenhui Chen, Qingling Yang, Linli Hu, Mengchen Wang, Ziyao Yang, Xinxin Zeng, Yingpu Sun

**Affiliations:** ^1^ Center for Reproductive Medicine, The First Affiliated Hospital of Zhengzhou University, Zhengzhou, China; ^2^ Henan Key Laboratory of Reproduction and Genetics, The First Affiliated Hospital of Zhengzhou University, Zhengzhou, China; ^3^ Henan Provincial Obstetrical and Gynecological Diseases (Reproductive Medicine) Clinical Research Center, The First Affiliated Hospital of Zhengzhou University, Zhengzhou, China

**Keywords:** PCOS, RIF (Recurrent Implantation Failure), integrated transcriptomic analysis, machine learning, TCA cycle

## Abstract

Polycystic ovary syndrome (PCOS) is a complex endocrine metabolic disorder that affects 5–10% of women of reproductive age. The endometrium of women with PCOS has altered immune cells resulting in chronic low-grade inflammation, which attribute to recurrent implantation failure (RIF). In this study, we obtained three PCOS and RIF datasets respectively from the Gene Expression Omnibus (GEO) database. By analyzing differentially expressed genes (DEGs) and module genes using weighted gene co-expression networks (WGCNA), functional enrichment analysis, and three machine learning algorithms, we identified twelve diseases shared genes, and two diagnostic genes, including GLIPR1 and MAMLD1. PCOS and RIF validation datasets were assessed using the receiver operating characteristic (ROC) curve, and ideal area under the curve (AUC) values were obtained for each disease. Besides, we collected granulosa cells from healthy and PCOS infertile women, and endometrial tissues of healthy and RIF patients. RT-PCR was used to validate the reliability of GLIPR1 and MAMLD1. Furthermore, we performed gene set enrichment analysis (GSEA) and immune infiltration to explore the underlying mechanism of PCOS and RIF cooccurrence. Through the functional enrichment of twelve shared genes and two diagnostic genes, we found that both PCOS and RIF patients had disturbances in metabolites related to the TCA cycle, which eventually led to the massive activation of immune cells.

## 1 Introduction

Polycystic ovarian syndrome (PCOS), one of the most common endocrine-reproductive-metabolic disorders in women, is characterized by chronic anovulation, hyperandrogenism, and polycystic ovarian morphology and is consistently associated with obesity, insulin resistance (IR), and low-grade chronic inflammation (1, 2). A variety of immune disorders often accompany PCOS patients, which are associated with infertility and consequently, impact ovarian function, oocyte quality, and endometrial receptivity (ER) (3). Especially in obese patients with high estrogen and androgen levels, dysfunction of immune cells may lead to continuous stimulation of the immune system, increasing pro-inflammatory cells (4). This pro-inflammatory condition can negatively affect critical physiological processes, such as proliferation, migration, and invasion of trophoblastic cells into the endometrium that ultimately lead to embryo implantation failure (5, 6). As a result, PCOS accounts for a nonnegligible part of the gynecological diseases that lead to recurrent implantation failure (RIF) during IVF/ICSI-ET (7).

RIF is defined as a lack of clinical pregnancy after at least four embryos have been transferred in a minimum of three fresh or frozen cycles in a couple under 40 years of age, with an incidence rate of about 15% (8). To date, many studies have shown that RIF etiology is largely attributed to three categories: decreased endometrial receptivity (ER), embryonic defects, and other combined effects (9). Among them, immune factors may play a crucial role in influencing ER and embryo implantation (10, 11). Normal immunological function of the maternal-fetal interface is critical for maintaining ER, which requires interactions with decidual cells, endothelial cells, and infiltrating immune cells (12, 13). These immune cells regulate maternal and fetal antigen responses, trophoblast invasion, and vascular remodeling (14). Recently, several transcriptomic studies of gene and protein expression profiles in the endometrium also identified some immune-related markers in RIF patients (15, 16). Significant progress has been made in search of the mechanism of RIF. However, RIF remains a common and insurmountable adverse event in assisted reproductive medicine.

Furthermore, the mechanism of impaired endometrial receptivity (ER) has attracted research interest in recent years (17, 18). Notably, in patients with PCOS, gene expression profiles are dysregulated (19, 20), with differentially expressed genes closely related to steroid hormone synthesis, inflammation, and oxidative stress (21). The establishment of ER depends on these biological processes. Moreover, some indicators are closely associated with ER (22), such as leukemia inhibitory factor (LIF) (23), homeobox genes A (HOXA), αvβ3-integrin, and intercellular junctions (24). As verified by clinical data analysis, damaged ER leads to an increased risk of repeated implant failure in PCOS patients (25). Poor endometrial receptivity is commonly identified as the primary cause of RIF, and abnormal gene expression contributes to ER deficiency (26). Therefore, the bioinformatics background of PCOS women may contain the underlying mechanism of recurrent implantation failure in this population.

In this study, we aimed to explore the potential biomarkers and underlying pathways involved in the development of PCOS and RIF. To achieve this goal, we integrated transcriptomic data related to PCOS and RIF from the Gene Expression Omnibus (GEO) and applied “LIMMA” package and weighted gene co-expression network analysis (WGCNA) to identify differentially expressed genes and critical modules in each disease. Our analysis using the intersection and three machine learning methods led to the identification of two disease-shared diagnostic genes, GLIPR1 and MAMLD1, with good performance validated by external datasets. Further, we conducted gene set enrichment analysis (GSEA) on each of these two genes to identify common pathways associated with PCOS and RIF. Additionally, we investigated the role of immune cells in the co-pathogenesis of the two diseases through immune infiltration analysis. Our results suggested that the co-pathogenesis of PCOS and RIF might be linked to increased immune response levels arising from abnormal TCA cycle metabolism. In conclusion, this study provides valuable insights into the shared molecular mechanisms underlying PCOS and RIF and highlights the potential of GLIPR1 and MAMLD1 as diagnostic markers for these conditions.

## 2 Methods

### 2.1 Data collection and preparation

**Figure 1:**
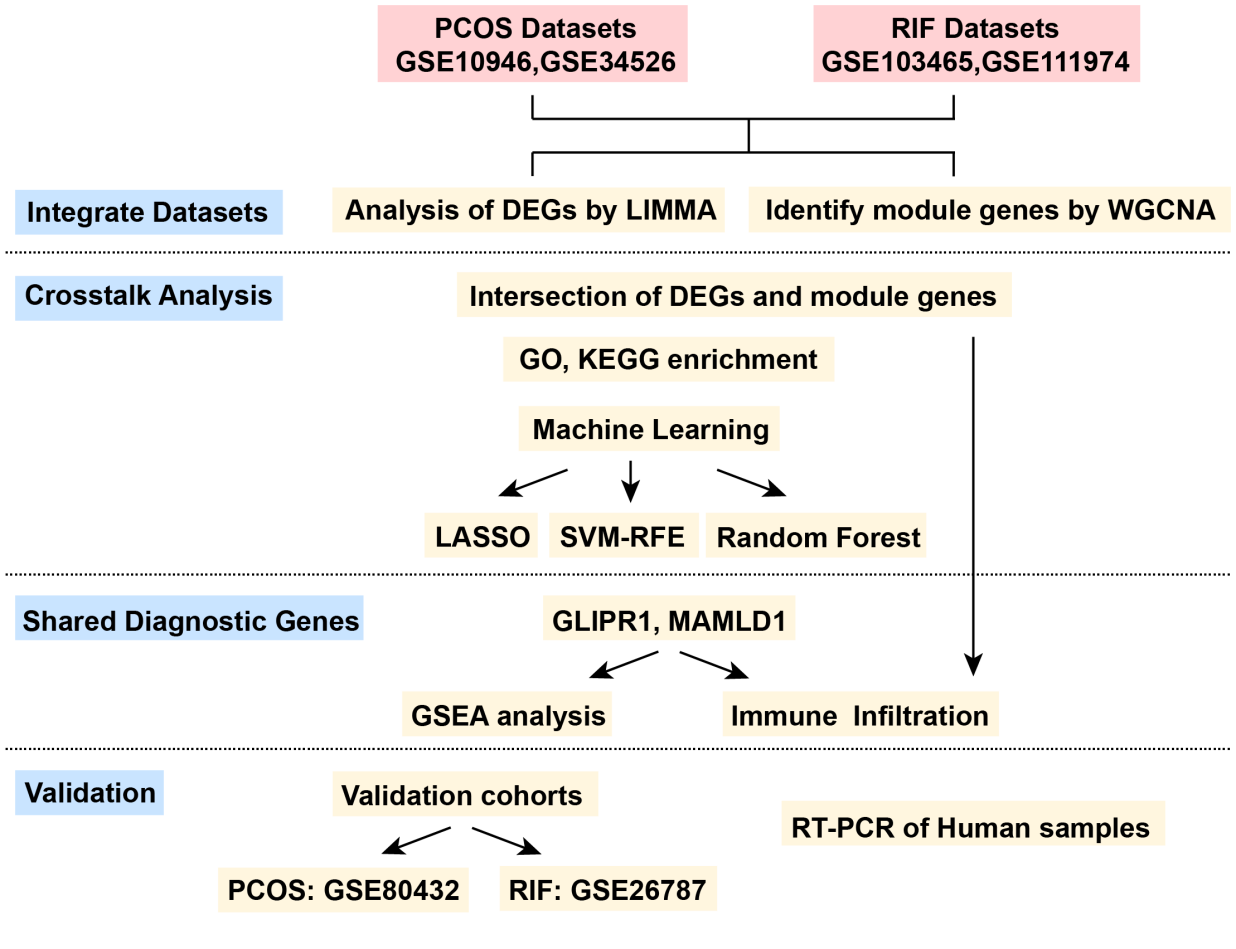
The flow chart for the whole design. PCOS, Polycystic Ovarian Syndrome; RIF, Recurrent Implantation Failure; GSE, Gene Expression Omnibus Series; LIMMA, Linear Models for Microarray Data; WGCNA, Weighted Gene Co-expression Network Analysis; DEGs, Differentially Expressed Genes; LASSO, Least Absolute Shrinkage and Selection Operator; SVM-RFE, Support Vector Machine- Recursive Feature Elimination; GSEA, Gene Set Enrichment Analysis.

The data sets related to PCOS and RIF were screened in the Gene Expression Omnibus database (GEO) (http://www.ncbi.nlm.nih.gov/geo/) since both diseases were designed in this study. For PCOS, we used the keyword “PCOS” or “granulosa cells” to search gene expression profiles. Inclusion criteria were as follows (1): PCOS patients and normal controls must be included in the profiles (2), The discovery profile should have at least ten samples to ensure accuracy, and (3) granulosa cells should be used for sequencing. Accordingly, we selected three datasets numbered GSE10946, GSE34526, and GSE80432. For RIF, the eligibility criteria were as follows (1): the profiles must include normal controls and the RIF patients, and (2) the sample source must be endometrial tissues. After the screening, the datasets GSE103465, GSE11974, and GSE26787 were included in this study. Among them, GSE10946 and GSE34526 of PCOS, and GSE103465 and GSE111974 of RIF were used as discovery cohorts for analyzing and screening. GSE80432 and GSE26787 were used for external validation for PCOS and RIF, respectively. The whole analytic workflow is shown in **Figure 1**.

Besides, when preparing the two datasets for each disease, the PCA plot showed a noticeable batch effect in the two disease groups. Thus, using the “sva” R package, which identified and built surrogate variables for high-dimensional data sets, the batch effect was eliminated. After removing the batch effect, the PCA plot was visualized by the “FactoMineR” and “Factoextra” R packages.

### 2.2 Differential gene expression analysis

After preparing the data for each disease, we compared PCOS and RIF datasets using the Linear Models for Microarray (LIMMA) package in R (version 4.1.2). Differentially expressed genes (DEGs) were calculated between disease and control groups with it. For PCOS, the DEG threshold was set as *P* value <0.05 and |log2FC(fold change)|>0.585. For RIF, the *P* value adjusted to 0.05 and |log2 FC|>1 were used to identify the DEGs. Next, the difference analysis results for each group were presented using the heatmap and volcano plot. In both plots, blue indicated low expression, and red indicated high.

### 2.3 Weighted gene co-expression network analysis (WGCNA)

Using microarray specimens, WGCNA represents one of the most important and widely applied systems bioinformatics methods to describe the correlation patterns among genes. Genes can be grouped into modules based on their co-expression similarities across samples using the “WGCNA” R package. Additionally, the WGCNA method can be used to connect modules to clinical elements outside the genome. In this way, relevant functional networks can be used to identify biomarkers and new molecules. As input files, normalized mRNA expression data (calculated using the R package “LIMMA”) were used to perform WGCNA to identify gene coexpression and the correlation between gene modules and clinical characteristics (PCOS or RIF compared to control groups). For each disease group, the following steps are followed (1): by using the R package “gplots,” hierarchical clustering analysis was performed to identify outliers in the sample (2), the “pickSoftThreshold” package function was utilized to screen out soft-power parameters ranging from 1 to 20 (3), a topological overlap matrix (TOM) is created by converting the matrix of correlations with the most appropriate β value to an adjacency matrix and then into a topological overlap matrix (4), based on the average linkage hierarchical clustering, an hierarchical clustering tree (linked gene best fit) was constructed, and then the dynamic tree cut algorithm (minModuleSize = 30) was used to find different gene modules. Similar modules were merged by a cuthight in each group, and (5) gene modules and clinical phenotypes (CTRL and PCOS or RIF) were correlated using the Pearson correlation coefficient. For PCOS, the highest correlation module antiquewhite4 was selected, and the genes associated with it were further analyzed. However, many modules were related strongly to RIF, so we further filtered the genes according to the gene significance (GS) and modular membership (MM). The disease was most closely related to genes with a high MM and GS, and we selected the genes in the hub modules with |MM|>0.8 and |GS|>0.5 for the RIF group.

### 2.4 Identification of shared genes and functional enrichment analysis

By combining DEGs and module genes identified by WGCNA, we were able to identify the shared key genes that contributed to the pathogenesis of both PCOS and RIF. For identifying genes’ biological functions and signaling pathways, we used the “clusterProfier” package for Gene Ontology (GO) and Kyoto Encyclopedia of Genes and Genomes (KEGG) pathway enrichment analysis. The bar plots showed the significant enrichment results of the functional enrichment with *P*<0.05.

### 2.5 Feature selection by three well-established machine learning algorithms

Further screening of co-existing genes between the two diseases was carried out using three well-established machine learning algorithms (LASSO: Least Absolute Shrinkage and Selection Operator; SVM-RFE: Support Vector Machine- Recursive Feature Elimination; RF: Random Forest). To ensure the repeatability of these algorithms, we set the seed at 123 in both disease groups.

Firstly, the 12 shared genes obtained previously were input into the LASSO algorithm in each disease group. We constructed a regression model using the R package “glmnet” with 10-fold cross-validation. In the “family” parameter, we set “binomial,” and we chose the best lambda value by “lambda.1min”. Logarithm (lambda) profiles of the LASSO coefficients were drawn for the 12 features. Then we drew the partial likelihood deviation (binomial deviation) curve and the logarithm (λ) curve. Next, we calculated the best value for 1se (1-SE standard) of minimum standard.

Following that, SVM-RFE was used to eliminate recursive features. Using the “e1071” and “MSVM-RFE” package for SVM modeling, SVM-RFE applied sequential backward feature elimination to determine the optimal hub gene. All of the 12 shared genes were used in our SVM model. The result of SVM-RFE was visualized, and by tenfold cross-validation, the red circle indicated maximum classification precision and the corresponding gene sets were the most accurate diagnostic markers at the lowest 5×CV error and the highest 5×CV accuracy.

Finally, we used Random Forest to classify the significant genes with the R package “randomForest”. Using a decision tree algorithm, random forest analysis identified which variables were most important. As a result of this algorithm, we were able to filter the shared genes to find disease signature genes. Our first step was to construct a random forest model using 500 trees on the discovery cohorts and determine the optimal number of trees using cross-validation errors. Then, we ranked genes by importance and plotted the 10 most significant genes. For each disease group, the significance threshold was set at 0.9 to decide the final result.

After screening the above three algorithms, we took the intersection of the results of each algorithm. There were five genes common to the PCOS group and seven common genes in the RIF group shown in a Venn plot. Furthermore, we took the intersection of the above common genes again and finally obtained two genes as the disease diagnostic target genes. The “pROC” package was used to construct ROC curves and displayed using “ggplot2” to assess the accuracy of the two diagnostic genes in discovery cohorts.

### 2.6 Prediction performance in validation cohorts

To further test the accuracy of the two diagnostic genes, we searched the GSE80432 for PCOS and GSE26787 of RIF for external validation. We first downloaded the raw data of these two data sets from the GEO database and normalized them with the “RMA” package. For GSE80432, we filtered out a deviation sample through PCA and finally adopted four healthy control samples and three PCOS patients. For GSE26787, five healthy controls and five patients with RIF were used for validation. The diagnostic gene expression pattern in validation cohorts was shown by boxplot, and AUC (area under the ROC curve) was also calculated.

### 2.7 Implementation of GSEA for single diagnostic gene

After obtaining the diagnostic genes, we performed single-gene gene set enrichment analysis (GSEA) for each diagnostic gene in the two groups using the “clusterProfiler” package. Using GSEA, we compared the biological signaling pathways between the disease group and the healthy control group. MSigDB (c5.go.bp.v7.5.1.entrez.gmt) was used to download gene sets. Enrichplot was used to show the top 5 activating and inhibiting pathways for each gene in the two disease groups.

### 2.8 Immune cell abundance

Each sample of disease was subjected to CIBERSORT analysis to determine the relative levels of immune cells. The CIBERSORT algorithm resolves immune cell composition by deconvolution based on gene expression data. According to CIBERSORT’s website (http://cibersort.stanford.edu/), LM22 contains 22 annotated gene signatures. With CIBERSORT and 1000 iterations, we quantified 22 types of immune cells based on the LM22 gene signature. In the following analysis, we selected samples with a CIBERSORT *P* value less than 0.05 for analysis. For each sample, the output estimates from CIBERSORT were normalized to sum to one to facilitate comparisons across immune cell types and datasets. R packages “corplot,” “vioplot” and “ggplot2” were used to visualize the results. Further, the correlation between immune infiltrated cells and diagnostic target biomarkers was determined by nonparametric correlations (Spearman).

### 2.9 Human samples collection

Granulosa cells of PCOS and the control patients were collected from infertile women who underwent ART treatment in the Reproductive Medicine Center of the First Affiliated Hospital of Zhengzhou University in China. Inclusion criteria for PCOS patients were based on the 2003 Rotterdam Criteria (27). Mid-secretory endometrial samples from the RIF and control groups were also collected to verify our data analysis. This study was approved by the Ethics Committee of the First Affiliated Hospital of Zhengzhou University in China. RNA of these samples was extracted using TRIzol Reagent (Invitrogen™, Japan) and cDNA was synthesized using HiScript III RT SuperMix for qPCR (Vazyme, Nanjing, China). Then real-time PCR (RT-PCR) was performed to quantitative the expression level of *GLIPR1* and *MAMLD1* in the two diseases. Primer sequences of the two genes were described in **Supplementary Table 1.** Statistical analysis was performed to compare the groups, using Student’s t-test. The data were presented as mean values accompanied by the standard error of the mean (SEM). A significance level of *P*<0.05 was considered a significant difference.

## 3 Results

### 3.1 GEO information

A total of four data sets were selected for discovery analysis according to our inclusion criteria: GSE10946, GSE34526, GSE103465, and GSE111974. **Table 1** summarized the detailed information on the four datasets. Among them, GSE10946 and GSE34526 were used as discovery cohorts for the PCOS, and GSE103465 and GSE111974 were regarded as discovery cohorts of RIF. Besides, GSE80432 and GSE26787 were the validation cohort of PCOS and RIF, respectively. In the present study, we called these two disease groups PCOS and RIF for short.

**Table 1:** Details of GEO datasets used in the study.

Diseases	GEO Series	GPL Platform	Sample Size		Group
			Control	Case	
PCOS	GSE10946	GPL570	11	12	Discovery cohort
	GSE34526	GPL570	3	7	Discovery cohort
	GSE80432	GPL6244	4	4	Validation cohort
RIF	GSE103465	GPL16043	3	3	Discovery cohort
	GSE111974	GPL17077	24	24	Discovery cohort
	GSE26787	GPL570	5	5	Validation cohort

PCOS, Polycystic Ovarian Syndrome; RIF, Recurrent Implantation Failure; GEO, Gene Expression Omnibus. Regarding GSE10946, we used both lean and obese non-PCOS samples as controls to avoid bias and reflect real-world metabolic diversity.

### 3.2 Identification of DEGs.

**Figure 2:**
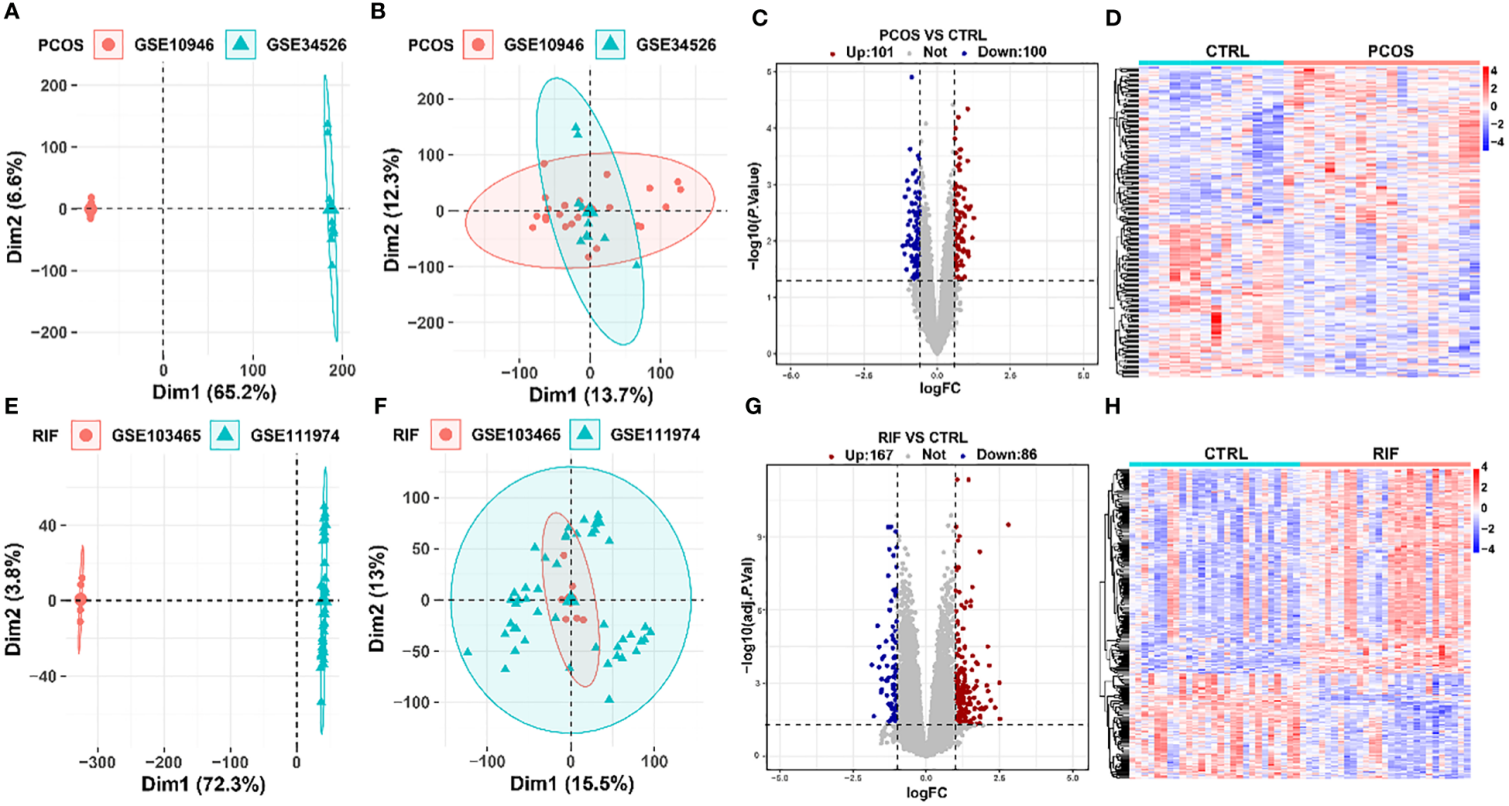
Removal of batch effects and identification of DEGs in PCOS and RIF. **(A, B)**. PCA plots showed the expression pattern in two datasets of PCOS before and after eliminating the batch effects. **(C, D)**. DEG heatmap and volcano plot in PCOS group. **(E, F)**. PCA plots showed the expression pattern in two datasets of RIF before and after removing the batch effects. **(G, H)**. Heatmap and the volcano plot of DEGs in RIF group. CTRL, Control; RIF, Recurrent Implantation Failure; PCA, Principle-component Analysis.

Before the biological information analysis, we tested the batch effects of the collected datasets and found that the batch effects of the two diseases were apparent (**Figure 2A, E**). Using the “sva” package, we removed the batch effects of the PCOS (**Figure 2B**) and RIF groups (**Figure 2F**) to obtain reliable analysis results. A LIMMA package was then used to characterize the DEGs between the two groups. 201 DEGs (*P*<0.05, |log2 FC|>0.585) with 101 up-regulated and 100 down-regulated genes were obtained for PCOS. For RIF, there were 253 DEGs (adj. *P*<0.05, |log2 FC|>1) with 167 up-regulated and 86 down-regulated genes. Volcano plots showed all DEGs of PCOS (**Figure 2C**) and RIF (**Figure 2G**) groups. Taken as a whole, the DEGs contained in the two groups were visualized by heatmaps (**Figure 2D, H**). DEGs associated with PCOS and RIF might play a role in their occurrence and development.

### 3.3 Screening for key modules by WGCNA.

**Figure 3:**
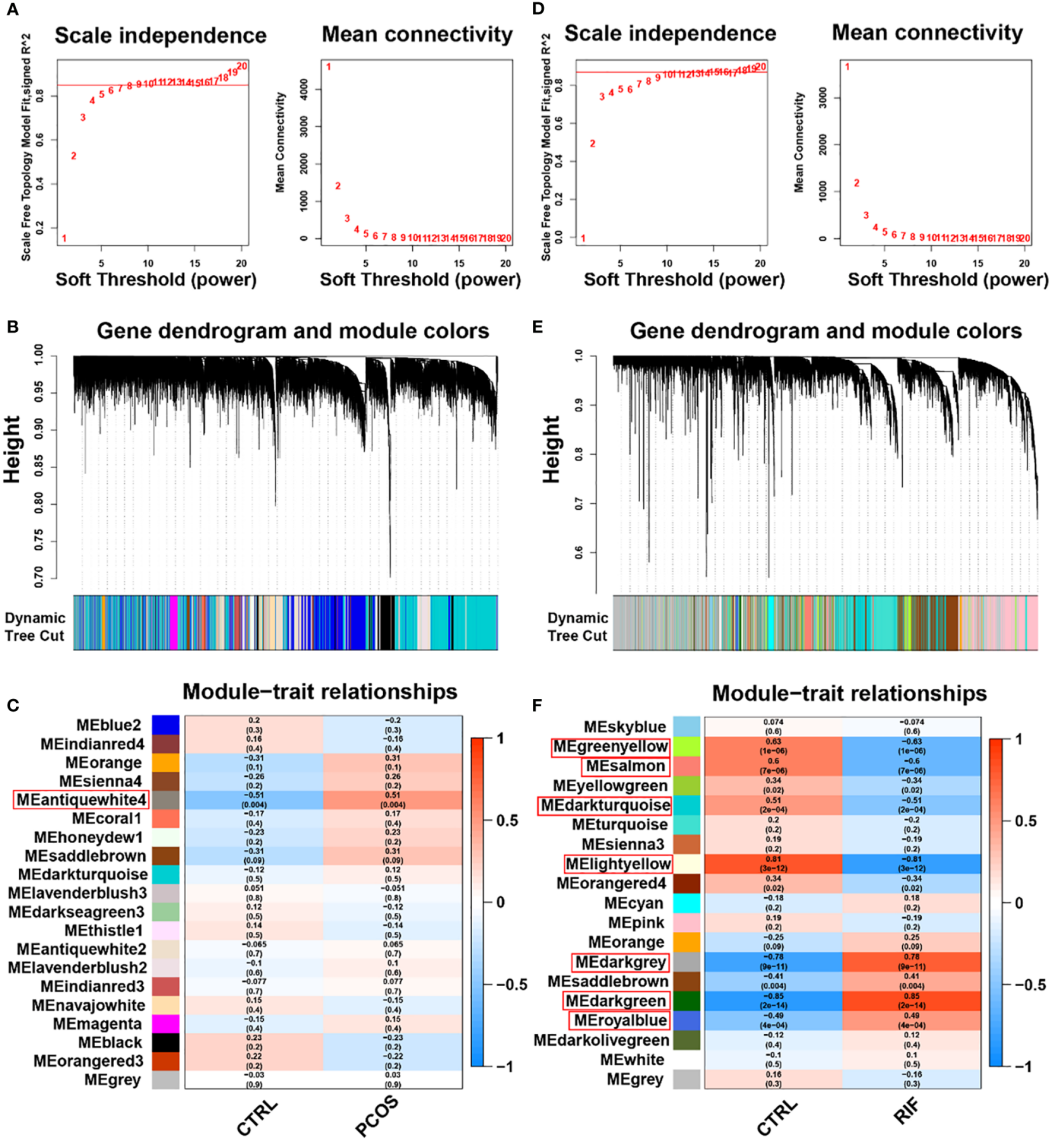
Weighted gene co-expression network analysis (WGCNA) of PCOS and RIF. **(A)**. Determination of soft-threshold power for PCOS. **(B)**. Cluster dendrogram of PCOS highly connected genes in key modules. **(C)**. Relationships between modules and traits in PCOS. Correlations and *P* values are included in each cell. **(D)**. Calculation of soft-threshold power for RIF. **(E)**. Cluster dendrogram of RIF modules with highly connected genes. **(F)**. Module–trait relationships in RIF. A correlation and *P* value are included in each cell. CTRL, Control; RIF, Recurrent Implantation Failure.

To investigate whether the diseases and key genes are correlated, we performed WGCNA in addition to analyzing the differential expression between the two groups. Using the soft-thresholding approach, this study constructed a co-expression network. This parameter β was essential for co-expression networks to maintain a scale-free topology. Gene expression data-based biological networks were most likely to be scale-free. Accordingly, in the PCOS group, the fit index greater than 0.85 was considered scale-free topology, and β was set at 9 (**Figure 3A**). By using the adjacency function, the adjacency matrix was generated. As shown in **Figure 3B**, hierarchical clustering was constructed using the TOM dissimilarity measure. We have identified 20 co-expression modules in total. The modules that *P*<0.05 were regarded as key modules. As shown in **Figure 3C**, the antiquewhite4 module had the strongest positive correlation, which contained 180 genes. Also, WGCNA was applied to the RIF group, and β=10 was the optimal value for soft power (**Figure 3D**). We identified 19 modules in total, in which dark grey, dark green, and royal blue showed a strong positive correlation, and green-yellow, salmon, dark turquoise, and light yellow modules showed a strong negative correlation (**Figures 3E, F**). Among the genes in these 7 key modules in the RIF group, we further selected 334 genes with |MM| > 0.8 and |GS| > 0.5. These genes in key modules derived from the two groups could be potentially used as candidate cell-type-specific markers.

### 3.4 Analysis of the shared genes and functional enrichment

**Figure 4:**
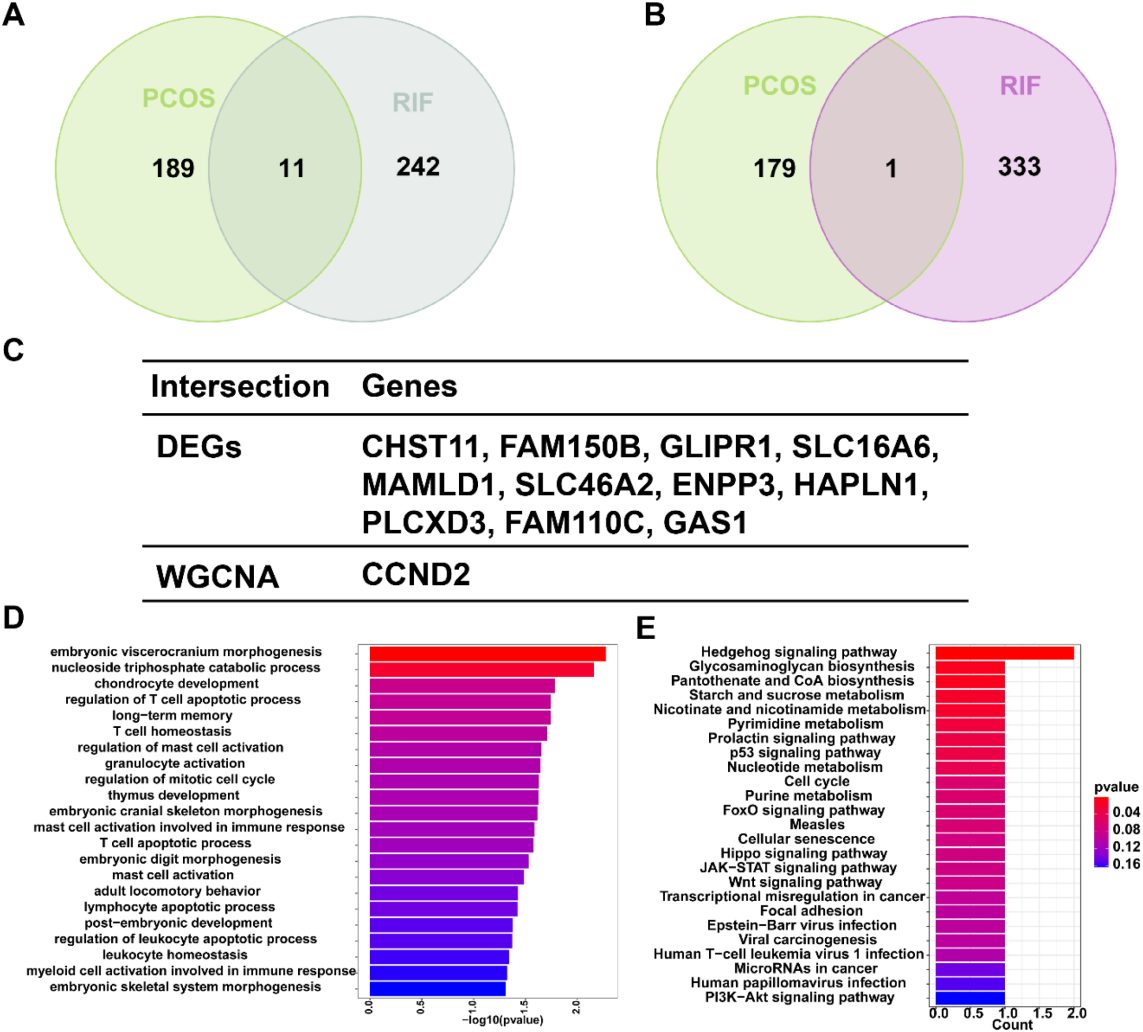
Shared gene signatures and functional enrichment between PCOS and RIF. **(A)**. The shared DEGs between PCOS and RIF by overlapping the DEGs of them. **(B)**. The shared genes between the WGCNA modules of PCOS and RIF by overlapping them. **(C)**. Table showed details of the shared genes. **(D, E)**. Shared genes were represented by bar plots displaying GO and KEGG enrichment. CTRL, Control; RIF, Recurrent Implantation Failure; GO, Gene Ontology; KEGG, Kyoto Encyclopedia of Genes and Genomes.

To explore the co-pathogenesis of PCOS and RIF, we took the intersection of the DEGs mentioned above and genes screened by WGCNA, respectively. **Figure 4A** showed an overlap between the DEGs of PCOS and RIF as a total of 11 genes (CHST11, FAM150B, GLIPR1, SLC16A6, MAMLD1, SLC46A2, ENPP3, HAPLN1, PLCXD3, FAM110C, GAS1). There was only one gene that overlapped the genes of WGCNA analysis (CCND2, **Figure 4B**). We speculated that these 12 genes might be related to the pathogenesis of PCOS and RIF and had a shared relationship (**Figure 4C**). Analyzing these genes for functional annotation and enrichment (**Figures 4D, E**), we sought to investigate the potential biological changes between PCOS and RIF. Not surprisingly, GO analysis of the shared genes revealed they were overrepresented in pathways associated with early embryonic organ morphogenesis. Notably, among all the GO terms enriched, we noticed that several significantly enriched biological processes pathways, such as regulation of T cell apoptotic process, mast cell activation, and leukocyte homeostasis, suggesting that the activation and apoptosis of immune cells might make a considerable contribution to the co-pathogenesis of PCOS and RIF. Besides, KEGG enrichment was consistent with the GO analysis. In addition, we noted that several pathways related to follicular development are enriched, such as the p53 signaling pathway, FOXO signaling pathway, hippo signaling pathway, and PI3K-Akt signaling pathway.

### 3.5 Identify potential shared diagnostic genes based on machine learning algorithms.

**Figure 5:**
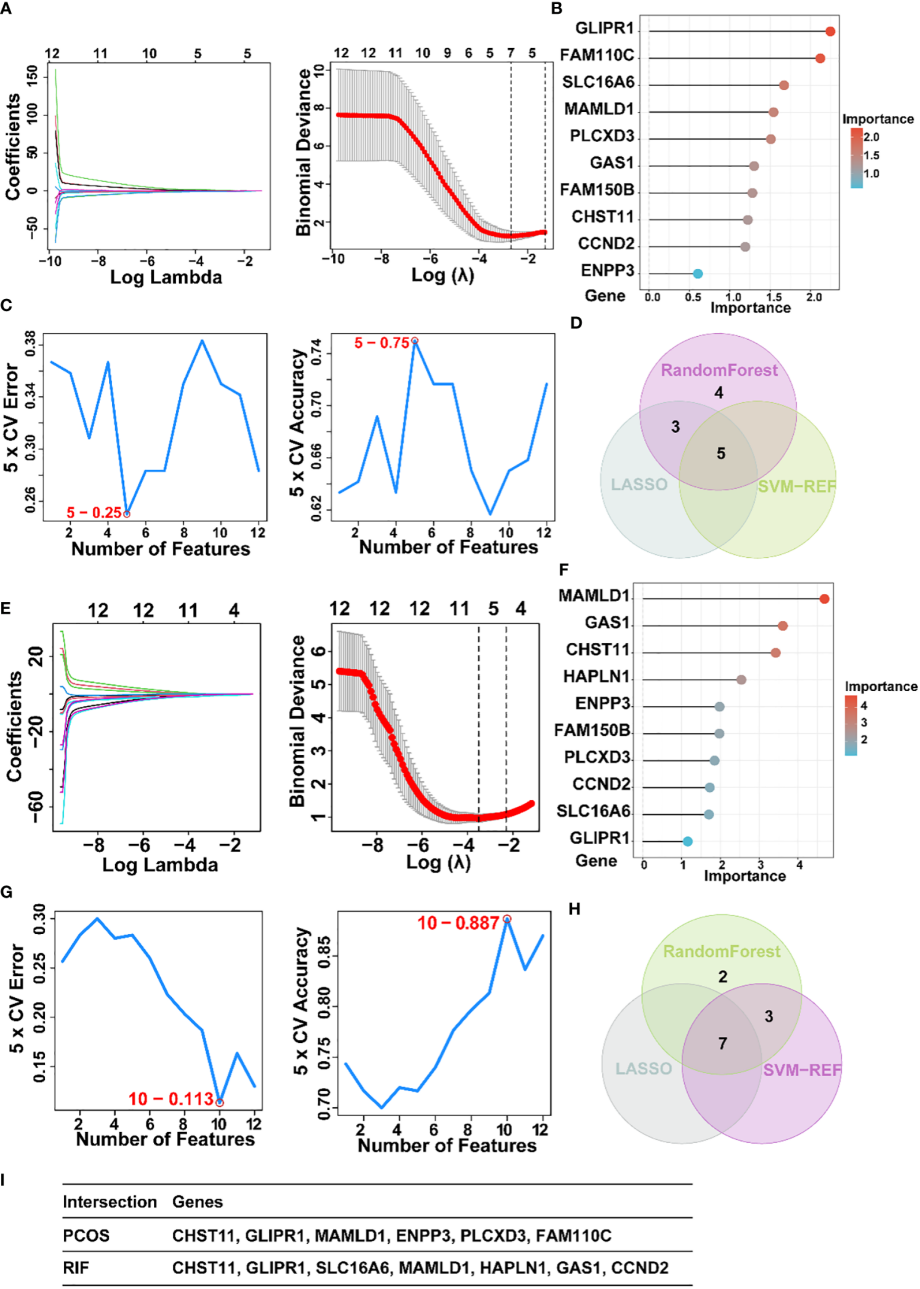
The screening of candidate PCOS and RIF diagnostic genes using three machine learning algorithms. **(A)**. Coefficient profile plot of the LASSO model for PCOS showed the final parameter selection λ (lambda). **(B)**. PCOS top-10 genes according to their discriminant ability in the RF algorithm. **(C)**. Five crosstalk genes were selected by using the SVM-RFE algorithm for PCOS. **(D)**. The Venn diagram showed five candidate diagnostic genes in PCOS by intersecting the results of three algorithms. **(E)**. Coefficient RIF profile plot of the LASSO model showed the selection of the optimal parameter λ (lambda). **(F)**. Top-10 RF algorithm step discriminant ability genes for RIF. **(G)**. Ten crosstalk genes were selected by using the SVM-RFE algorithm for RIF. **(H)**. The Venn diagram showed seven candidate diagnostic genes in RIF by intersecting the results of three algorithms **(I)**. Table showed the details of candidate diagnostic genes in PCOS and RIF. CTRL, Control; RIF, Recurrent implantation failure; LASSO, Least Absolute Shrinkage and Selection Operator; SVM-RFE, Support Vector Machine-Recursive Feature Elimination; RF, Random Forest.

For a further selection of the most candidate diagnostic gene targets with a significantly characteristic value of classifying the disease groups and control groups, three different algorithms (LASSO, SVM-RFE, and Random Forest) were applied based on the above 12 shared genes. In the PCOS group, based on the LASSO coefficient profiles and the optimal tuning parameter selection map, λ was set at 0.06851194 (PCOS) (**Figure 5A**). Afterward, eight genes with non-zero coefficients were found. Then we inputted the above 12 genes into the RF classifier, and the top 10 genes were shown on the importance scale. We selected 0.9 as the screening threshold of importance, and a set of 9 genes was identified (**Figure 5B**). Also, the SVM algorithm identified 5 genes with the lowest 5-point CV error and best 5-point CV accuracy (**Figure 5C**). Furthermore, by overlapping these three algorithms, we developed 5 shared biomarkers (CHST11, GLIPR1, SLC16A6, MAMLD1, HAPLN1, GAS1) for the PCOS group (**Figures 5D, I**).

Similarly, 7 featured genes were obtained for the RIF group when the λ was set at 0.03001025 by the LASSO algorithm (**Figure 5E**). **Figure 5F** showed the top 10 genes on the importance scale and we chose 9 genes (importance>0.9) as the RM result. Next, a subset of 10 hub genes were identified using the SVM-REF algorithm (**Figure 5G**). Then, 7 common gene biomarkers obtained by the three algorithms overlapped (**Figures 5H, I**).

### 3.6 Diagnostic value and validation of diagnostic hub biomarkers.

**Figure 6:**
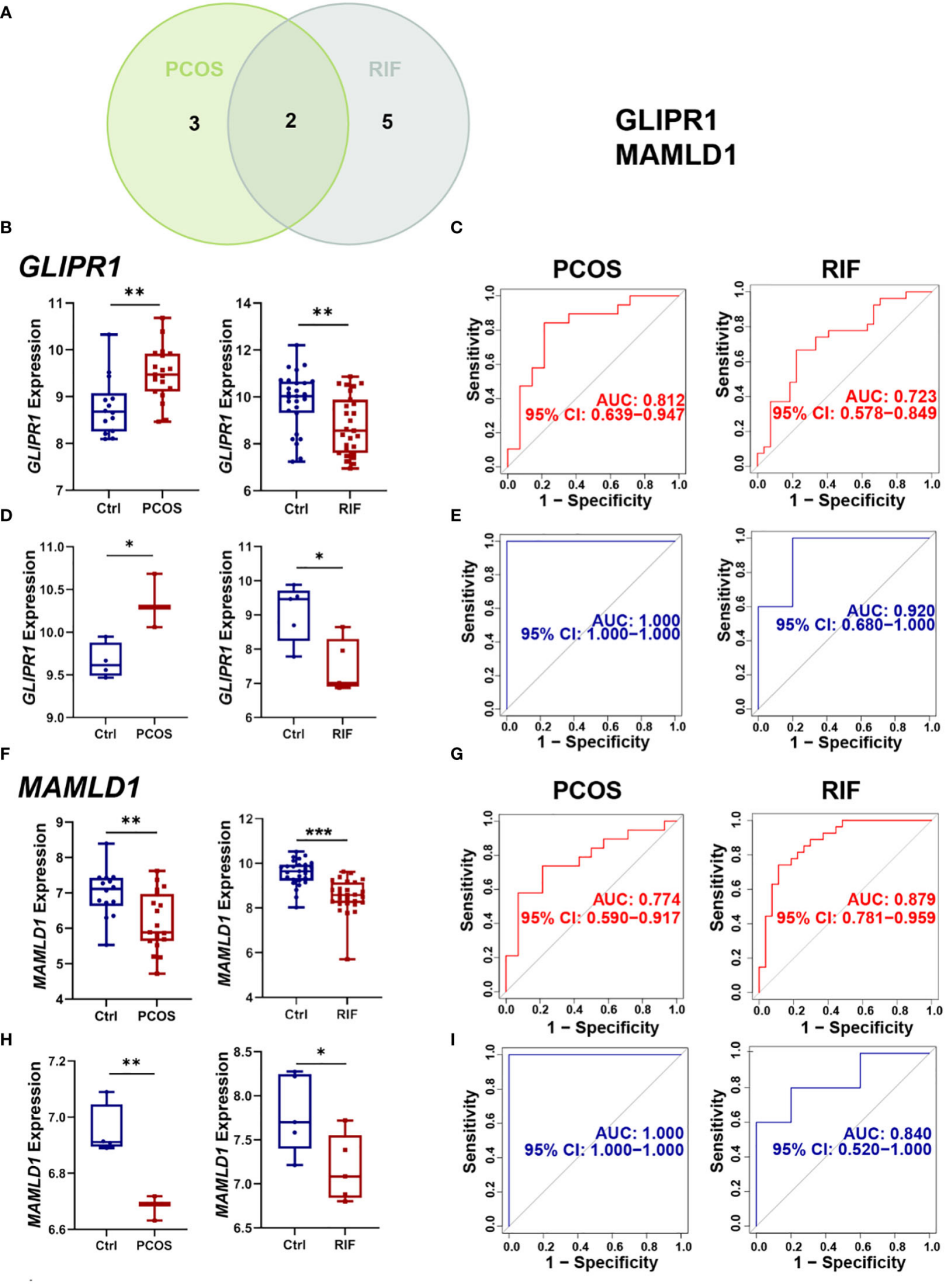
Selection and validation of the two shared diagnostic genes. **(A)**. The Venn plot showed the two shared diagnostic genes. **(B)**. Differential expression of GLIPR1 in the training group for PCOS and RIF. **(C)**. ROC curve of GLIPR1 in the training group for PCOS and RIF. **(D)**. Differential expression of GLIPR1 in the validation group for PCOS and RIF. **(E)**. ROC curve of GLIPR1 in the validation group for PCOS and RIF. **(F)**. Differential expression of MAMLD1 in the training group for PCOS and RIF. **(G)**. ROC curve of MAMLD1 in the training group for PCOS and RIF. **(H)**. Differential expression of MAMLD1 in the validation group for PCOS and RIF. **(I)**. ROC curve of MAMLD1 in the validation group for PCOS and RIF. ROC, receiver operating characteristic. *P< 0.05, **P< 0.01, ***P< 0.001.

For a more precise understanding of the relationship between PCOS and RIF, we took the intersection of machine learning results in the PCOS and RIF group and got 2 shared diagnostic genes, GLIPR1 and MAMLD1 (**Figure 6A**). And the prediction and the discriminatory ability of the shared diagnostic genes were assessed by analyzing the expression pattern of the two genes. Also, an analysis of the receiver operating characteristic curves (ROC curves) was conducted.

Firstly, we analyzed the PCOS and RIF expression levels of the two discovery cohorts. **Figure 6B** showed that *GLIRP1* was lower in the RIF groups (*P*<0.01) and higher in the PCOS groups (*P*<0.01). *MAMLD1* expressed lower both in the PCOS (*P*<0.01) and RIF groups (*P*<0.0001) (**Figure 6F**).

Next, to test the specificity and sensitivity of the two target genes for the diagnosis of two diseases, ROC analysis was applied. In terms of PCOS biomarkers, these two genes had favorable results: GLIPR1(AUC=0.812), and MAMLD1(AUC=0.774). The RIF group was also subjected to the same ROC analysis. Predictive performance was robust for each biomarker: GLIPR1(AUC=0.723), and MAMLD1(AUC=0.879) (**Figures 6C, G**).

Moreover, we confirmed the reliability of GLIPR1 and MAMLD1 as core diagnostic genes for PCOS and RIF by conducting external validation. In the two validation groups, the expression levels of the two hub genes matched those in the discovery cohorts. *GLIPR1* was decreased (*P*<0.05) in the RIF group and increased in the PCOS groups (*P*<0.05) (**Figure 6D**). *MAMLD1* was reduced both in the PCOS groups (*P*<0.01) and the RIF groups (*P*<0.05) (**Figure 6H**). **Figure 6E** showed that GLIPR1 had excellent diagnostic accuracy in the validation cohort of PCOS (AUC=1.000) and RIF (AUC=0.920). Similarly, MAMLD1 also properly diagnosed PCOS (AUC=1.000) and RIF (AUC=0.840) (**Figure 6I**). As a result, the results confirmed their ability to serve as key discriminatory molecules for PCOS and RIF, respectively.

### 3.7 Single-Gene GSEA of diagnostic genes.

**Figure 7:**
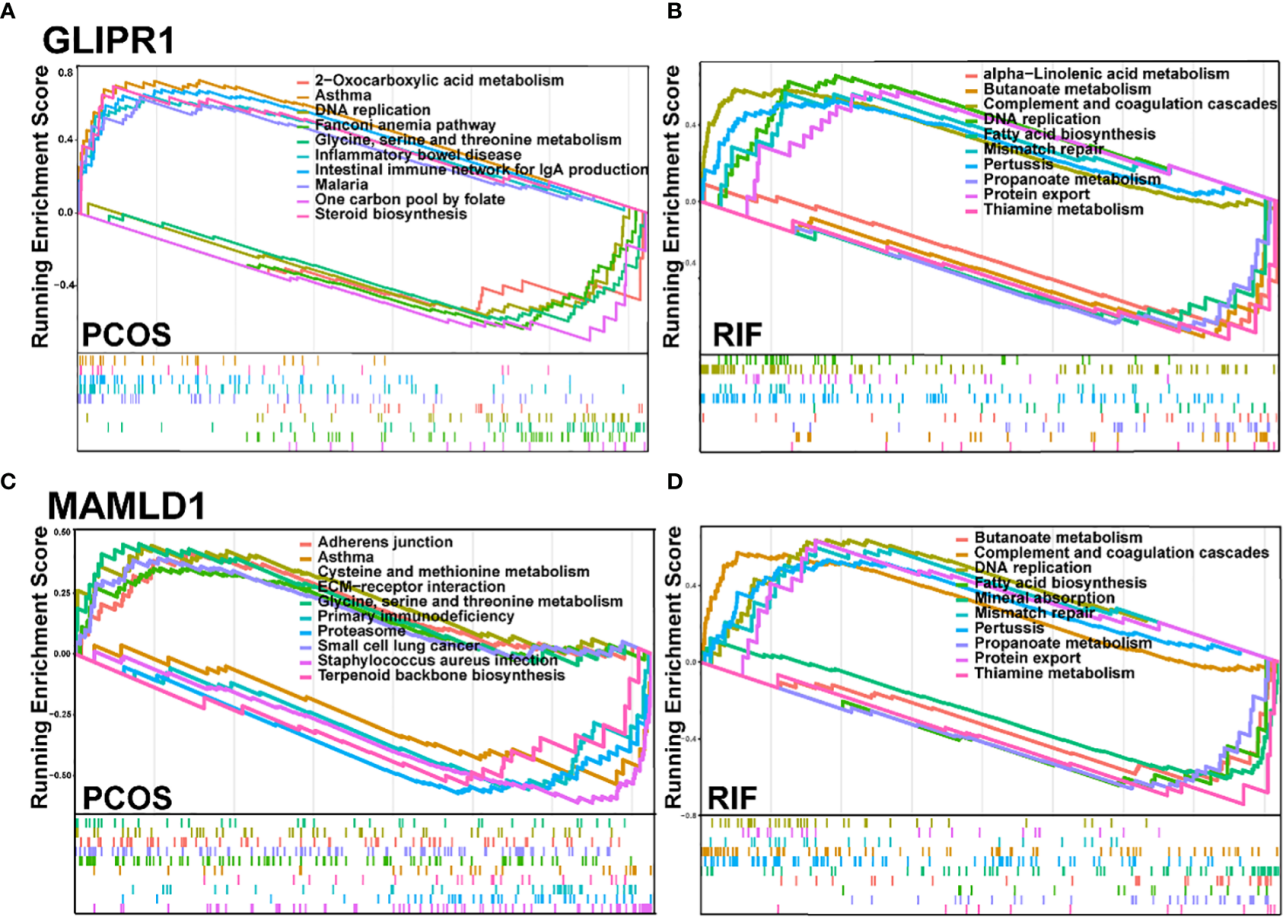
GSEA for the single diagnostic gene**. (A, B)**. GSEA analysis for GLIPR1 in PCOS and RIF group. **(C, D)**. GSEA analysis for MAMLD1 in PCOS and RIF group. GSEA, Gene Set Enrichment Analysis.

Subsequently, we employed single-gene GSEA analysis of the two biomarkers in PCOS and RIF datasets, respectively, and the top 5 up and down-regulated pathways were visualized by the “GSEA” package. **Figure 7** showed that in both disease groups, these two genes were both involved in metabolic pathways such as glycine, serine, threonine metabolism, alpha-linolenic acid metabolism, and propanoate metabolism. Besides, both genes enriched in inflammation-related pathways which linked the PCOS and RIF.

### 3.8 Immune infiltration analysis of shared diagnostic genes.

**Figure 8:**
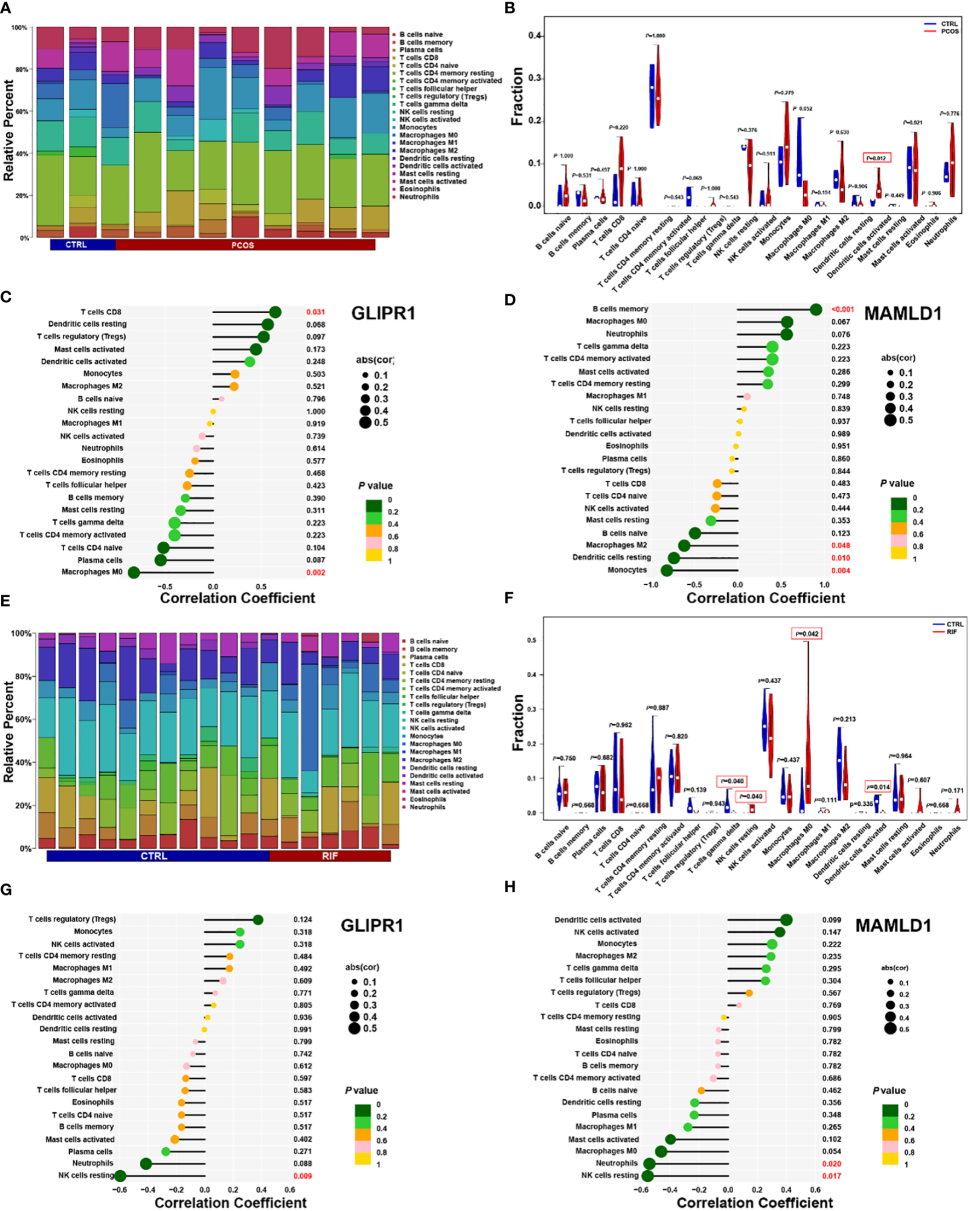
PCOS and RIF immune cell composition. **(A)**. Infiltrating immune cells were plotted in a stacked bar chart for the PCOS group. **(B)**. Violin diagram indicated that the PCOS group exhibited a significantly different type of immune cell. **(C)**. Correlation between GLIPR1 expression and immune cells in the PCOS group. **(D)**. Relationship between the expression of MAMLD1 and immunity in PCOS patients. **(E)**. Stacked bar chart showed the RIF group’s characteristics of infiltrating immune cells. **(F)**. Evident difference in immune cell types shown by violin diagram of the RIF group. **(G)**. Detection of GLIPR1 expression in immune cells in the RIF group. **(H)**. Relationship between MAMLD1 expression and immune cells in the RIF group. *P* < 0.05 was highlighted.

Considering that PCOS and RIF are characterized by a high immune response. The abundances of immune cells in different groups were analyzed with CIBERSORT. In each group, the proportion of 22 immune cells was shown as a bar plot. Generally, the bar graphs clearly illustrate the significant differences between the percentages of T cells, macrophages, and NK cell populations between PCOS (**Figure 8A**) and RIF (**Figure 8E**). Compared with the control samples, the dendritic cells activated were increased in the PCOS samples (**Figure 8B**). While, in the RIF samples, NK cells resting, Macrophages M0 were increased, and NK cells gamma delta, dendritic cells activated were decreased (**Figure 8F**).

Moreover, the relationship between biomarkers and immune cell contents was investigated. In PCOS samples, CD8 T cells were significantly positively correlated with GLIPR1 (**Figure 8C**). In contrast, macrophage M0 correlated negatively. MAMLD1 was significantly positively correlated with B cells memory and negative for macrophages M2, dendritic cells resting, and monocytes (**Figure 8D**). In RIF samples, GLIPR1 had a significant negative correlation with NK cells resting (**Figure 8G**). While Neutrophils and NK cells rested negatively with MAMLD1 (**Figure 8H**). It appears that immune function is crucial to the development of PCOS and RIF.

### 3.9 Validation of GLIPRand MAMLDby RT-PCR in human tissues.

**Figure 9:**
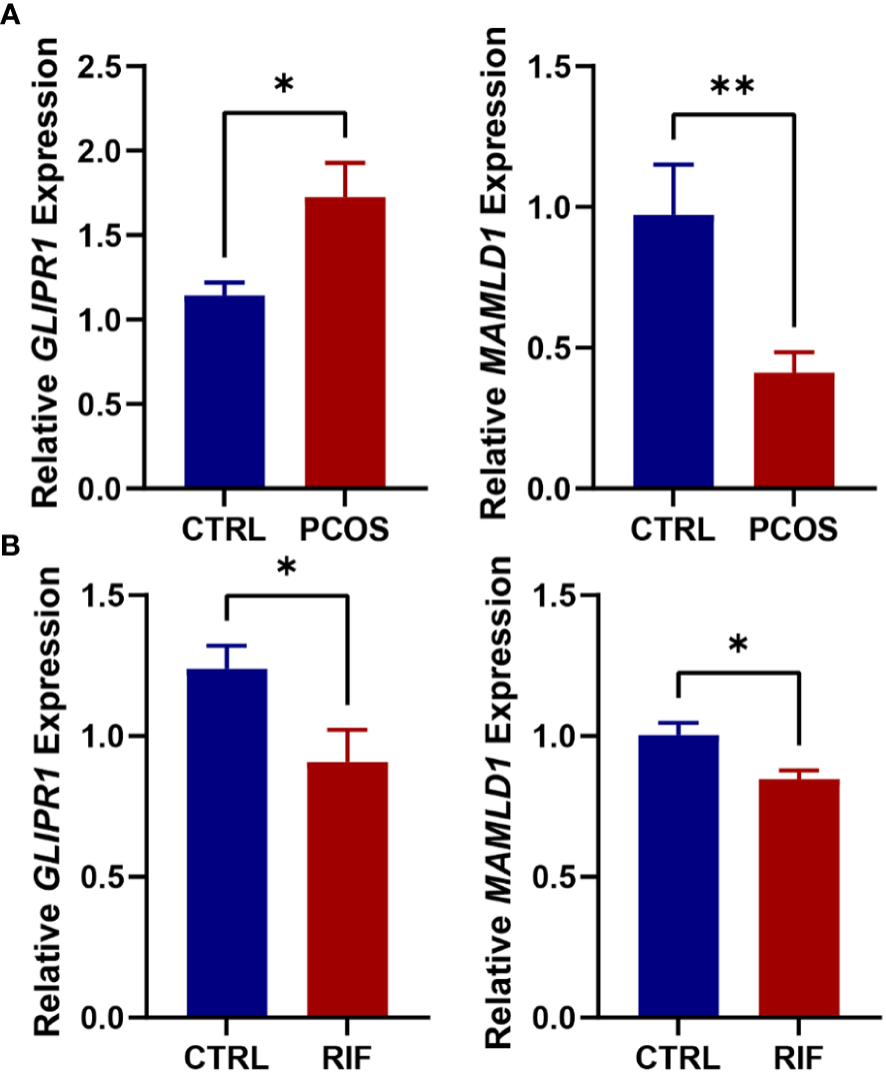
Validation of RT-PCR in human tissues. **(A)**. Expression levels of *GLIPR1* and *MAMLD1* in granulosa cells of normal and PCOS patients. (n=5) **(B)**. Expression levels of *GLIPR1* and *MAMLD1* in endometrial tissues of normal and RIF patients. (n=3) *P< 0.05, **P< 0.01.

RT-PCR was performed on follicular fluid-derived granulosa cells from normal women and PCOS patients and endometrial tissues from healthy and RIF women. This confirmed the gene expression levels of the two diagnostic biomarkers, *GLIPR1* and *MAMLD1*. Consistent with the data analysis, our results showed that *GLIPR1* expression was upregulated and *MAMLD1* expression was decreased in the granulosa cells of PCOS patients, (**Figure 9A**) while both *GLIPR1* and *MAMLD1* expression was reduced in endometrial tissues of RIF patients (**Figure 9B**).

## 4 Discussion

Due to the high occurrence of recurrent implantation failure for PCOS patients in ART cycles (8, 25), it has become essential to explore the common pathogenesis of the two diseases. In the present study, we applied the WGCNA (28) and three machine learning approaches to identify the common gene population and 2 diagnostic genes of GLIPR1 and MAMLD1 which were both significantly related to PCOS and RIF. Further, by GSEA (29) analysis, we uncovered that the co-pathogenesis of the two diseases lay in the abnormal metabolism of many metabolites associated with the TCA cycle, which led to the abnormal activation of immune cells and immune response in the disease group. Taken together, the newly discovered diagnostic genes and potential molecular mechanism in this study provided new clinical insights and guidance for diagnosing and treating PCOS and RIF patients.

GLI pathogenesis-related 1 (GLIPR1) is a gene that encodes a protein with diverse biological functions, including cell apoptosis, cell cycle regulation, and DNA damage response. While the direct relationship between GLIPR1 and PCOS and RIF has not been fully established, several studies have suggested a potential involvement of GLIPR1 in the pathogenesis of these diseases. Notably, altered expression levels of GLIPR1 have been observed in PCOS patients, indicating a possible association between GLIPR1 and PCOS (30). Studies have also identified that GLIPR1 encodes proteins that regulate sperm-oocyte binding and mature male germ cells, which have been linked to PCOS risk modification and metabolic mechanisms (31). Moreover, RNA sequencing of adipose tissue in PCOS patients has identified GLIPR1 to be differentially expressed according to genotype near PCOS risk loci (32), suggesting a possible relationship between insulin resistance and fertility. Single-cell mRNA sequencing of proliferative phase endometrial cells also identified GLIPR1 as having an important role in the perivascular environment (33). Additionally, GLIPR1 has been implicated in RIF. A study reported differential expression of GLIPR1 in RIF patients compared to those with successful pregnancies, suggesting its involvement in the immune response associated with embryo implantation (34). Our study suggested that GLIPR1 represented a potential diagnostic target for RIF in PCOS patients which could be supported by previous research partly.

MAMLD1 (NM_001177465) is a gene associated with disorders of sexual development in 46, XY individuals, and it has been reported that mutations in MAMLD1 resulted in male fetal sexual development (35) (36, 37). Furthermore, MAMLD1 hypomethylation and upregulation of its transcript in granulosa cells have been suggested as contributing to PCOS ovaries with excess androgen and hormone disbalance (38). Many studies have proved that the excess of maternal androgen leads to abnormal placental morphogenesis and impaired endometrial receptivity (39), resulting in granulosa cell apoptosis and inhibition of proliferation. Consequently, decreased ER ability and maternal-fetal interface eventually led to embryo implantation failure. Additionally, knock-out mice models have shown that lack of MAMLD1 causes parturition failure, high neonatal mortality rates, and an adverse effect on functional luteolysis (40). Consistent with previous studies, our analysis revealed that MAMLD1 appeared to play a role in embryo development and was associated with recurrent implantation failure in women with PCOS.

To further explore the underlying pathogenic association and mechanisms between PCOS and RIF, we performed GSEA for the two diagnostic genes in two disease groups. As a result of GSEA analysis, both PCOS and RIF genes were enriched in metabolism pathways. In the PCOS group, both two genes were enriched in glycine, serine, and threonine metabolism. Similarly, GLIPR1 and MAMLD1 were found to involve in the metabolism of butanoate, propanoate, and thiamine in the RIF group. Interestingly, metabolites like glycine (41), serine (42), and threonine (43) have been reported to contribute to increasing protein motive force, which is attributed to the activation of the TCA cycle and mitochondrial respiration chain. A previous study found that GLIPR1 influenced the energetic potential of mitochondria which was in accordance with these enrichment analyses (44). Specifically, butyrate has been reported to provide energy substrates for the host, which enter into the TCA cycle as acetyl-CoA to produce glucose (45). Propanoate can also increase the level of mitochondrial CoA and promote energy production through the TCA cycle (46). Known as vitamin B1, thiamine was used in the TCA cycle to form thiamine diphosphate (ThDP) (47). These metabolism changes all suggest us metabolites involved in the TCA cycle are disrupted in patients with PCOS complicated by repeated implantation failure and finally lead to the imbalance of energy metabolic homeostasis.

Consistent with previous studies (48, 49), our data showed that the common gene population mainly enriched in the activation pathways of immune cells like T cells, NK cells, macrophages, and neutrophils. These results hinted us the high level of immunity in PCOS and RIF patients and prompted us to further investigate which immune cells are abnormal in the two diseases. As anticipated, by immune infiltration (50), we detected Dendritic cells (DCs) activated in PCOS samples and four immune cell types in RIF samples, including T cells gamma delta, NK cells resting, macrophages M0 and Dendritic cells activated with significantly different abundance compared to healthy samples. The obesity status of PCOS patients will lead to changes in the number of DCs, and high androgen and low progesterone status will reduce the ability to recruit NK cells (4, 51). So that the secretion of cytokines is not enough to maintain maternal immune tolerance, and eventually leads to endometrial receptivity impairment, abortion, implantation failure, and other adverse events (52). While normal human pregnancy requires complex coordination between maternal immune tolerance and homeostasis (53). Besides, we uncovered that the prevalence of DCs in each disease was significantly different from the normal group. This suggests that the abnormal function of NCs may be the main cause of repeated implantation failure in PCOS patients.

Based on the above findings, we hypothesize that activation of a large number of immune cells in cellular immunity caused by disturbances of metabolites associated with the TCA cycle accounts for the co-pathogenesis of the PCOS and RIF. As previously mentioned, activated DCs and macrophages showed a truncated TCA cycle that resulted in an accumulation of citrate. Of note, the TCA cycle intermediates may leak from impaired mitochondria, and increasing evidence indicated that these metabolites of the TCA cycle played a substantial role in immune regulation (54). In PCOS patients with coexisting metabolic disorders, disruptions to their mitochondrial membranes may result in the release of TCA cycle intermediates into the cytosol, thereby impairing the cellular immune system. The dysregulated immune system is characterized by a large number of activated immune cells, particularly DCs, as observed in the present study. These processes ultimately contribute to the failure of embryo implantation, which is a hallmark feature of RIF.

Some limitations need to be mentioned in the present study. First, only 3 datasets were selected for each disease, including 18 cases and 14 control samples in the PCOS group, and 27 cases in both the RIF disease and normal groups for discovery. For WGCNA and immune infiltration analysis, more sufficient samples are needed to ensure test accuracy. Second, we know little about the processing of the raw data, and the datasets used in our study were profiled by microarray which was far behind today’s advanced sequencing technology. These intrinsic traits of these public datasets pose a challenge to the accuracy and advancement of our analysis.

In conclusion, diagnostic biomarkers GLIPR1 and MAMLD1 were identified as critical biomarkers responsible for regulating immune cell activation caused by an imbalance of TCA cycle metabolites. Our analysis reinforces the theoretical basis for the co-pathogenesis of recurrent pregnancy failure in PCOS patients.

## Data availability statement

The original contributions presented in the study are publicly available. Publicly available datasets used in this study can be downloaded from the GEO database (http://www.ncbi.nlm.nih.gov/geo/). The PCOS group includes three datasets with GSE10946, GSE34526, and GSE580432. GSE103465, GSE111974, and GSE26787 are involved in the RIF group. Further inquiries can be directed to the corresponding authors.

## Ethics statement

The studies involving human participants were reviewed and approved by the Ethics Committee of Scientific Research and Clinical Trial of the First Affiliated Hospital of Zhengzhou University. Written informed consent for participation was not required for this study in accordance with the national legislation and the institutional requirements.

## Author contributions

WC and QY conceived the study idea. WC performed the data analysis and produced the results. YS, QY, and WC wrote and revised the manuscript. LH, WC, MW, ZY, and XZ were responsible for sample collection and experiment. All authors contributed to the article and approved the submitted version.
