# Correction: Shared diagnostic genes and potential mechanism between PCOS and recurrent implantation failure revealed by integrated transcriptomic analysis and machine learning

**DOI:** 10.3389/fimmu.2025.1682282

**Published:** 2025-08-25

**Authors:** Wenhui Chen, Qingling Yang, Linli Hu, Mengchen Wang, Ziyao Yang, Xinxin Zeng, Yingpu Sun

**Affiliations:** ^1^ Center for Reproductive Medicine, The First Affiliated Hospital of Zhengzhou University, Zhengzhou, China; ^2^ Henan Key Laboratory of Reproduction and Genetics, The First Affiliated Hospital of Zhengzhou University, Zhengzhou, China; ^3^ Henan Provincial Obstetrical and Gynecological Diseases (Reproductive Medicine) Clinical Research Center, The First Affiliated Hospital of Zhengzhou University, Zhengzhou, China

**Keywords:** PCOS (polycystic ovarian syndrome), RIF (Recurrent Implantation Failure), integrated transcriptome analysis, machine learning, TCA cycle

There was an error in **Table 1** as published. The GSE103465 dataset includes a total of 6 samples, consisting of 3 controls and 3 patients. We inadvertently wrote “6 patients,” which was a typographical error. We sincerely apologize for this oversight. Our subsequent analyses were performed using the correct data from GSE103465 (3controls and 3 patients). This correction does not affect any part of the analysis or the conclusions drawn from the data. The corrected **Table 1** appears below.

**Table 1:** Details of GEO datasets used in the study.

Diseases	GEO Series	GPL Platform	Sample Size		Group
			Control	Case	
PCOS	GSE10946	GPL570	11	12	Discovery cohort
	GSE34526	GPL570	3	7	Discovery cohort
	GSE80432	GPL6244	4	4	Validation cohort
RIF	GSE103465	GPL16043	3	3	Discovery cohort
	GSE111974	GPL17077	24	24	Discovery cohort
	GSE26787	GPL570	5	5	Validation cohort

PCOS, Polycystic Ovarian Syndrome; RIF, Recurrent Implantation Failure; GEO, Gene Expression Omnibus. Regarding GSE10946, we used both lean and obese non-PCOS samples as controls to avoid bias and reflect real-world metabolic diversity.

Also, there was an error in **Figure 4C** as published. We apologize for the typographical error in **Figure 4C** — the gene symbol was mistakenly written as “LCXD3” instead of the correct “PLCXD3”. The corrected **Figure 4C** appear below.

**Figure 4:**
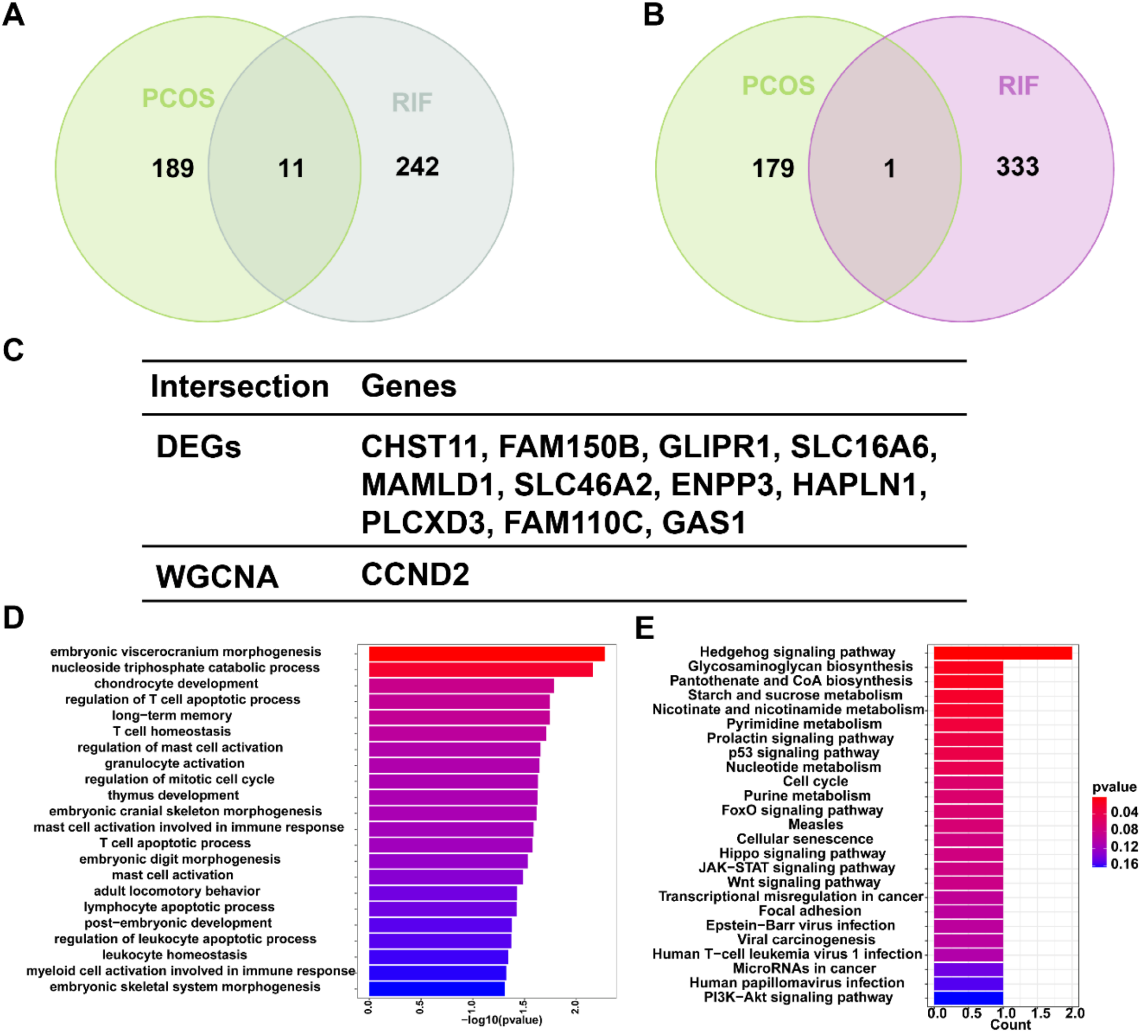
Shared gene signatures and functional enrichment between PCOS and RIF. **(A)** The shared DEGs between PCOS and RIF by overlapping the DEGs of them. **(B)** The shared genes between the WGCNA modules of PCOS and RIF by overlapping them. **(C)** Table showed details of the shared genes. **(D, E)** Shared genes were represented by bar plots displaying GO and KEGG enrichment. CTRL, Control; RIF, Recurrent Implantation Failure; GO, Gene Ontology; KEGG, Kyoto Encyclopedia of Genes and Genomes.

Lastly, there was an error in the legend for **Table 1** as published. The corrected legend appears below.

“TABLE 1 Details of GEO datasets used in the study. PCOS: Polycystic Ovarian Syndrome, RIF: Recurrent Implantation Failure, GEO: Gene Expression Omnibus. Regarding GSE10946, we used both lean and obese non-PCOS samples as controls to avoid bias and reflect real-world metabolic diversity.”

The original version of this article has been updated.

