# Supplementary material for: Shared diagnostic genes and potential mechanism between PCOS and recurrent implantation failure revealed by integrated transcriptomic analysis and machine learning
Source: Front Immunol. 2023 May 16;14:1175384. doi: 10.3389/fimmu.2023.1175384 (PMC10228695; doi:10.3389/fimmu.2023.1175384)
Supplement: Supplementary file 1 [file Table_1.docx]

**Supplementary Table 1. Primer sequences**

| **Gene** | **Forward primer** | **Reverse primer** |
| --- | --- | --- |
| ***GLIPR1*** | **ATGCGTGTCACACTTGCTACA** | **TCACCTCTGATCGGAACTTGT** |
| ***MAMLD1*** | **GGGAACTGTTAAGAGGAGACAAG** | **CCGCAGGGCTAGGATTTATTG** |
| ***GAPDH*** | **GCACCGTCAAGGCTGAGAAC** | **TGGTGAAGACGCCAGTGGA** |
